# Long-term cancer survivors treated with multiple courses of repeat radiation therapy

**DOI:** 10.1186/s13014-021-01934-y

**Published:** 2021-10-30

**Authors:** Sebastian M. Christ, Maiwand Ahmadsei, Lotte Wilke, Anja Kühnis, Matea Pavic, Stephanie Tanadini-Lang, Matthias Guckenberger

**Affiliations:** Department of Radiation Oncology, University Hospital Zurich, University of Zurich, Rämistrasse 100, 8091 Zurich, Switzerland

**Keywords:** Radiation therapy, Repeat irradiation, Multiple courses

## Abstract

**Introduction and background:**

Through recent advances in cancer care, the number of long-term survivors has continuously increased. As a result, repetitive use of local radiotherapy for curative or palliative indications might have increased as well. This analysis aims to describe patterns of care and outcome of patients treated with multiple courses of repeat radiotherapy.

**Materials and methods:**

All patients treated with radiotherapy between 2011 and 2019 at our department of Radiation Oncology were included into this analysis. A course of radiotherapy was defined as all treatment sessions to one anatomical site under one medical indication. Demographics, cancer and treatment characteristics and overall survival of patients having undergone multiple radiotherapy courses (minimum n = 5) were evaluated.

**Results:**

The proportion of cancer patients treated with a minimum five courses of radiotherapy increased continuously from 0.9% in 2011 to 6.5% in 2019. In the 112 patients treated with a minimum of five radiotherapy courses, the primary tumor was lung in 41.9% (n = 47), malignant melanoma in 8.9% (n = 10) and breast in 8.0% (n = 9) of cases. A median interval of 3 years (maximum 8 years) elapsed between the first and the last radiotherapy course. The maximum number of courses in a single patient were n = 10. Treatment intent was curative or palliative in 46.4% and 53.6% for the first radiotherapy, respectively. The proportion of curative intent decreased to 11.6% at the 5th, and the last radiotherapy course was following a palliative intent in all patients. Five-year overall survival measured from the 1st radiotherapy course was 32.7%. Median overall survival was 3.3, 2.4, 1.3, and 0.6 years when measured from the 1st, the 1st palliative, the 5th and last course of radiotherapy, respectively.

**Discussion and conclusion:**

A continuously increasing number of patients is treated with multiple courses of radiotherapy throughout their long-term cancer survivorship.

## Introduction and background

Over the past 2 decades, cancer has started to evolve into a chronic condition. This development was driven by continuous advances in cancer care, especially on the medical oncological front in the form of new therapeutics and by technological advances in surgery and radiation oncology [1, 2]. The population of long-term cancer survivors therefore increased in developed countries [3]. This development forms the background for a potentially increasing use and frequency of radiotherapy (RT) during long-term cancer survivorship. Curative indications for repeat radiotherapy might be isolated loco-regional tumor recurrence, oligometastatic disease recurrence, late secondary cancer development and de-novo secondary malignancies. Moreover, despite the improved efficacy of modern targeted therapies and immune checkpoint inhibition, the majority of patients will ultimately develop drug resistance, with a potential need for repeat radiotherapy with palliative intent.

Despite these well-documented developments in oncology and despite a growing body of literature about patients treated with in-field re-irradiation once or twice, there is surprisingly no data in the published literature about long-term cancer survivors treated with *multiple* courses of RT. This lack of prospective and retrospective data about cancer patients treated with multiple courses of repeat radiotherapy refers to its patterns-of-care, safety, and efficacy. It is therefore the aim of this retrospective single-institution study to analyze patients treated with *multiple courses of repeat* RT during their chronic disease history as cancer patient.

## Materials and methods

### Patient cohort

All patients treated with RT between 2011 and 2019 at our Department of Radiation Oncology were included into this analysis and were screened for treatment with multiple RT courses. A course of RT was defined as a prescribed treatment to one anatomical site under the umbrella of one medical indication at one particular point in time in the patient history. Regarding the time dimension, for a RT to count as a new course, a minimum of 30 days needed to elapse since the last RT. For example, a patient having undergone RT for an oropharynx primary and for two lung metastases 6 months later, was counted as having received two courses of RT. However, a patient with small cell lung cancer (SCLC), who received RT to the primary and to synchronous brain metastasis, was counted as having received one course of RT only. The total number of RT courses was assessed in the Record and Verify System (Aria® Version 15, Varian®). We used the term *multiple repeat RT* (MRRT) to characterize a unique cohort of patients, who were treated with minimum five radiotherapy courses during their disease history. A minimum of five RT courses was chosen for inclusion into this study because of the lack of safety and efficacy data in the literature about such patients.

### Data collection

Demographic, cancer and treatment characteristics were extracted from the Record and Verify software ARIA® and the following parameters were available for analysis: General patient information (date of birth, gender), RT treatment characteristics (treatment intent, treatment site (classified according to the 10th edition of the international classification of diseases (ICD-10) code), RT start and end date, RT fractionation, single fraction dose and total RT dose and course count, and date of death for overall survival (OS) calculation as the primary endpoint of this study. The data was complemented with variables from two other sources: The ICD-10 primary diagnosis was obtained from the in-hospital tumor documentation system OnkoStar™ which was cross checked against the medical records system KISIM™, from which the date of primary diagnosis was manually extracted as well. The medical records were also used to cross-check and contextualize data extracted from the treatment planning system. Survival status was last updated on March, 1st 2021 by consulting the electronic patient file, publicly available death registries or by contacting the primary care physician in charge. This project was approved by the Swiss Cantonal Ethics Committee (BASEC# 2021-00104).

### Statistical analysis

Upon extraction of the data, Microsoft® Excel® (Version 16.0) was used to store, clean and assess the collected information. Summary statistics were subsequently computed using the statistical software package STATA® (Version 16.1.). The non-parametric Mann–Whitney test was used to evaluate differences between groups and statistical significance was set at *p* < 0.05. Kaplan–Meier OS was calculated from the time of the first RT course, the first palliative RT course, the 5th and the last RT course to date of death or last follow-up. Graphical displays were compiled employing the visualization software package GraphPad PRISM® (Version 8).

## Results

**Fig. 1:**
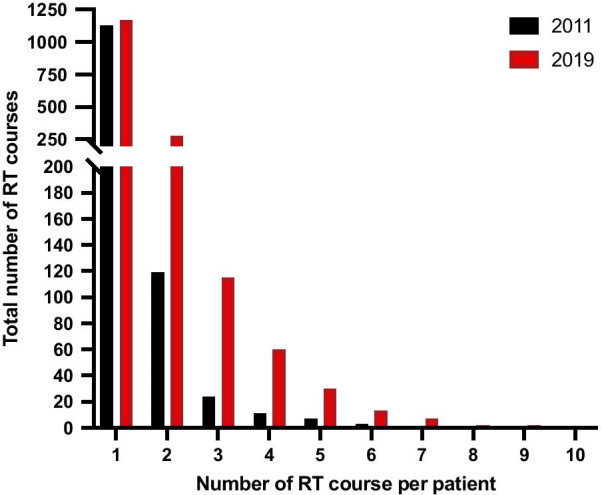
Overall distribution of the number of radiotherapy courses per patient. *RT* radiation therapy

In 2011, the proportion of patients treated with MRRT was 0.9% (12/1293) of all patients treated at our Department of Radiation Oncology. By 2019, this proportion of patients increased significantly to 6.5% (110/1674) (*p* value = 0.01). Figure 1 illustrates the overall distribution of the number of radiotherapy courses per patient treated in 2011 and 2019.

For the 112 patients treated with MRRT, the median age at primary diagnosis was 56 (range, 26–85) years. Approximately half of the patients were female (n = 51, 45.5%). More than 40% of patients had lung cancer (n = 47, 41.9%) and the 2nd and 3rd most frequent primary tumor histologies were malignant melanoma (n = 10, 8.9%) and breast cancer (n = 9, 8.0%). Eight (7.1%), seven (6.3%) and seven (6.3%) patients were treated for soft tissue/bone sarcoma, colorectal or head and neck cancers, respectively. Detailed patient characteristics are summarized in Table 1.

**Table 1:** Patient characteristics

Parameter	Data (n = 112 patients)
Age at primary diagnosis in years, median (range)	56 (26–85)
Female gender, n (%)	51 (45.5)
Primary tumor histology, n (%)	
Lung^a^	47 (41.9)
Malignant melanoma	10 (8.9)
Breast cancer	9 (8.0)
Soft tissue and bone	8 (7.1)
Colorectal	7 (6.3)
Head and neck	7 (6.3)
Other^b^	24 (21.4)
Alive at time of analysis, n (%)	31 (27.7)

^a^Includes non-small cell lung cancer (NSCLC), small-cell lung cancer (SCLC), and mesothelioma

^b^Includes prostate, urinary tract, endocrine, gynecologic, hematologic, esophageal and hepatocellular entities as well as cancer of unknown origin

The 112 patients were treated with a total of 660 RT courses. The median number of courses per patient was five (range, 5–10) and the number of patients treated with six to seven and eight to ten repeat RT courses was n = 39 (34.8%) and n = 13 (11.6%), respectively. The maximum number of RT courses in a single patient was 10 (n = 2, 1.8%). Median duration of one single RT course was 14 (1–97) days. A median of 3 (0–8) years elapsed between the 1st and the last RT course.

The three most common treatment sites were bone (n = 265, 40.1%), brain (n = 214, 32.4%) and lung (n = 71, 10.1%). Thirty-six (5.4%) RT courses targeted a primary tumor site. The median number of prescribed RT fractions was six (1–35), with a median single fraction dose of four (1.8–20) Gray (Gy), and a median total dose of 30 (3–70) Gy. Treatment-related data is summarized in Table 2.

**Table 2:** Treatment characteristics

Parameter	Data (n = 660 RT courses; n = 112 pts)
Number of RT courses, median (range)	5 (5–10)
Number of RT courses per patient	
5, n = patients (% of total patients)	60 (53.6)
6, n = patients (% of total patients)	25 (22.3)
7, n = patients (% of total patients)	14 (12.5)
8, n = patients (% of total patients)	8 (7.1)
9, n = patients (% of total patients)	3 (2.7)
10, n = patients (% of total patients)	2 (1.8)
Treatment duration in days, median (range)	14 (1–97)
Interval (years) between first and last radiotherapy course, median (range)	3 (0–8)
Number of radiotherapy fractions, median (range)	6 (1–35)
Dose per fraction in Gray, median (range)	4 (1.8–20)
Total dose in Gray, median (range)	30 (3–70)
Treatment intent	
Curative, n (%)	147 (22.3)
Palliative, n (%)	513 (77.7)
Treatment site	
Bone, n (%)	265 (40.1)
Brain, n (%)	214 (32.4)
Lung, n (%)	71 (10.1)
Primary, n (%)	36 (5.4)
Lymph nodes, n (%)	29 (4.4)
Liver, n (%)	16 (2.4)
Soft tissue, n (%)	13 (2.0)
Adrenals, n (%)	9 (1.4)
Other, n (%)^a^	7 (1.1)

*Pts* patients, *RT* radiation therapy

^a^Includes mediastinum, kidneys, thyroid and pleura

The treatment intent had been specified for every RT course individually by the treating radiation oncologist. The large majority of RT courses was prescribed with a palliative intent (n = 513, 77.7%). As expected, a significant trend to a palliative treatment intent was observed over the courses of RT. While treatment intent at the first course was curative in 46.4% and palliative in 53.6% of the cases, the treatment intent remained curative in only 25.9% at the 2nd and 18.8% at the 3rd RT course. Subsequently, treatment intent was curative or palliative in 11.6% and 88.4% at the 5th course, respectively. At 9th and 10th courses were performed with palliative intent (Fig. 4 in Appendix).

Regarding the timing of MRRT, the intervals between RT courses became shorter as the number of RT courses increased. For all 112 patients, the median interval between primary cancer diagnosis and the start of the first RT course was 8.2 months. Median interval between subsequent RT courses ranged between 6.8 and 1.7 months. The detailed treatment trajectories are illustrated in Fig. 5 in Appendix.

**Fig. 2:**
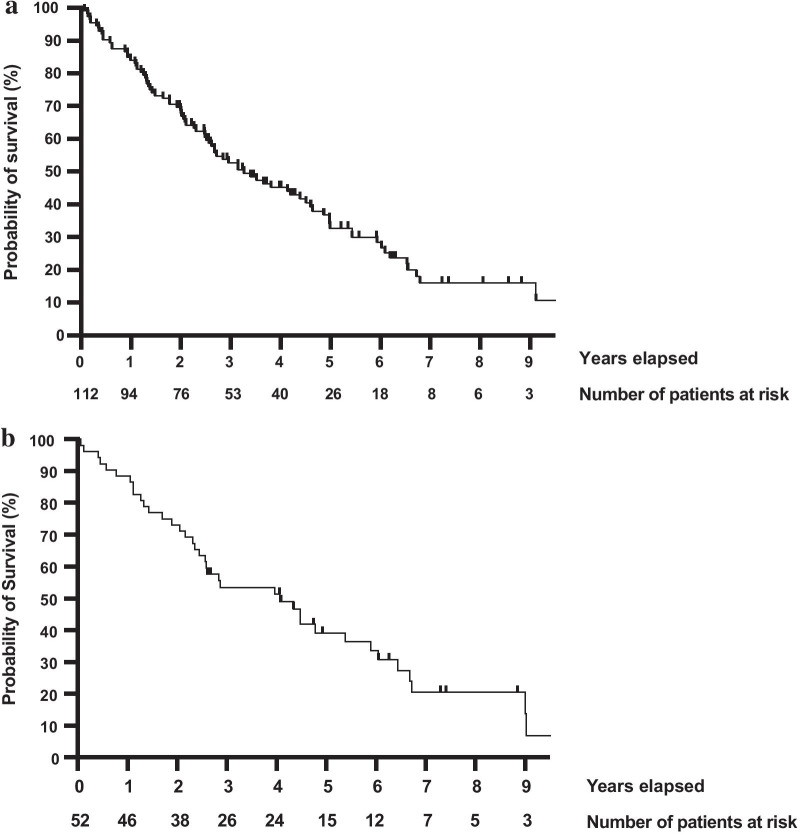
Overall survival from time of **a** 1st RT treatment course, **b** 1st curative RT course. *RT* radiation therapy

At the time of final data analysis in March 2021, 31 (27.7%) patients in this cohort were still alive (Table 1). The median OS measured from the first RT course was 3.3 years and 5-year OS was 32.7% (Table 3, Fig. 2a). Median OS measured from the first curative RT was 4.1 years and 5-year OS was 39.2% (Table 3, Fig. 2b). Median OS measured from the first palliative RT was 2.4 years and 5-year OS was 24.9%. Median OS measured from the 5th and last RT course were 1.3 years and 0.6 years, respectively. Survival statistics are compiled in Table 3.

**Table 3:** Overview of survival statistics

Parameter	Median OS	Median 5-year survival
From date of primary diagnosis, years; %	6.1	57.3
From 1st RT, years; %	3.3	32.7
From 1st curative RT, years; %	4.1	39.2
From 1st palliative RT, years; %	2.4	24.9
From 5th RT, years; %	1.3	15.7
From last RT, years; %	0.6	8.5

*OS* overall survival, *RT* radiation therapy

**Fig. 3:**
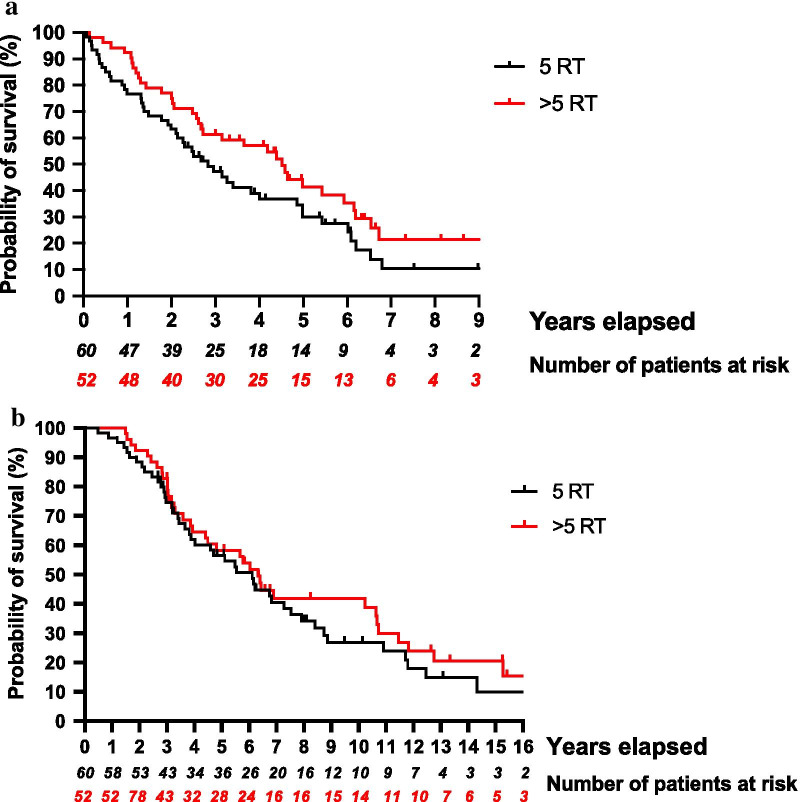
Comparison of overall survival from **a** 1st RT for patients with 5 RT versus > 5 RT courses, (*p* value = 0.07), **b** date of primary diagnosis for patients with 5 RT versus > 5 RT courses, (*p* value = 0.48). *RT* radiation therapy

**Fig. 4:**
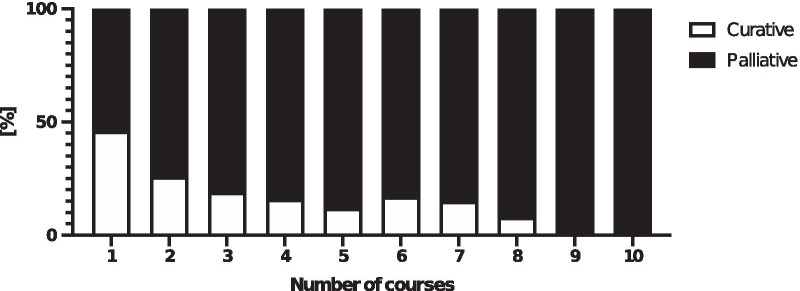
Treatment intent over the course of multiple RT treatment courses. *RT* radiation therapy. *Patients may be counted more than once. **The non-linear trend between palliative and curative intent is due to the “dynamic oligometastatic state model”

**Fig. 5:**
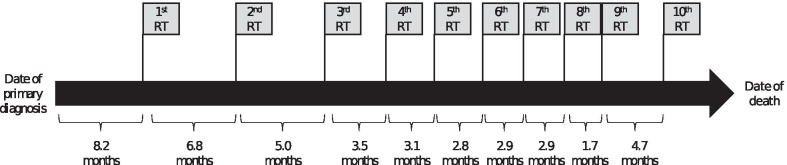
Treatment time between different RT courses at our department. *RT* radiation therapy. ^1^Median time between courses is calculated based on all patients having received up to the nth course

When comparing OS between patients treated with five versus more than five RT courses, the group of patients having undergone more than five RT courses was characterized by a longer OS measured from the time of first RT of median 2.8 vs 4.5 years, respectively; this difference was not statistically significant (*p* value = 0.073). OS measured from time point of primary cancer diagnosis was also not significantly different (Fig. 3a, b).

## Discussion

This is to the best of our knowledge the first study which analyzed patterns of radiotherapy care of cancer patients treated with multiple courses of repeat radiotherapy. Over the past decade, the cohort of patients treated with a minimum five courses of repeat radiotherapy has increased continuously in our department. In 2011, the proportion of patients treated with MRRT was 0.9%, which increased 6.5% in 2019. These patients were characterized by a long OS expectancy when measured from the first course of radiotherapy. However, median OS was still > 7 months from the last course of radiotherapy, indicating that patients had sufficiently long-life expectancy to achieve a potential benefit from all courses of repeat radiotherapy.

There is only a small body of literature assessing patients who received repeat RT which is compiled below. Muller et al. [4] studied a series of 44 patients with metastatic Non-small cell lung cancer (NSCLC), of whom seven patients underwent three or more courses of RT, for which favorable OS and progression-free survival (PFS) were reported. Devos et al. [5] assessed metastasis-directed therapy in 191 oligorecurrent prostate cancer patients, 25 of whom received three or more RT courses. The authors came to the conclusion that the approach is a feasible and a promising concept of care [5]. A study from Volpe et al. [6] examined a very small series of eight patients with prostate cancer, who were treated with two or three courses of repeat RT for locally recurrent disease; the authors concluded that multiple RT courses may present a salvage therapy option for selected patients with low tumor burden. Ogawa et al. [7] analyzed 31 patients with in-field local tumor relapses of NSCLC or lung metastasis and reported that repeat stereotactic body radiotherapy (SBRT) constitutes a viable therapeutic regimen. Further Fritz et al. [8] assessed the feasibility of repeat stereotactic radiosurgery (SRS) in 42 patients harboring 197 brain metastases with 16 patients having undergone minimum three courses of SRS. The authors concluded that repeat SRS constitutes a reasonable treatment option in selected patients to delay rescue whole-brain radiation therapy [8]. Barton et al. [9] conducted an epidemiological calculus in more than 60,000 patients to assess the retreatment rate in different radio-oncological facilities, and found that it was fairly similar. In summary, available studies focused on small numbers of selected patients with mostly lung and prostate cancer as well as brain metastases who received repeat RT. However, patients were mostly treated with maximum three or four courses of RT, without providing a more systematic and comprehensive research approach.

Of all 112 patients treated having received MRRT, lung cancer was by far the most frequent primary tumor site, contributing 41.9% of this cohort. This finding of rapidly increasing numbers of lung cancer patients treated with MRRT over the last 10 years corresponds to the tremendous advances in the field of medical oncology, specifically targeted therapy for NSCLC patients with activating driver mutations and immune checkpoint inhibition [10, 11]. Additionally, recent advances in imaging (e.g., fluorodeoxyglucose positron emission tomography (FDG-PET), diagnostics (e.g., endobronchial ultrasound), surgery (e.g., laparoscopic and robotic approaches) and radiotherapy (e.g., SBRT) have advanced the multidisciplinary care of lung cancer patients. Despite the fact, that prolonged life expectancy of lung cancer patients is predominantly the result of more effective systemic therapy, repeat RT appears to be playing an important role in the concept of cancer as a chronic disease. Beyond palliative intents aiming at symptom control or relief, ablative RT for oligoprogressive disease is practiced with increasing frequency [5, 12], despite the benefit of this concept not having been proven in a randomized study design. All patients in our center are regularly presented and discussed in the framework of multidisciplinary tumor boards, indicating the importance for further increased collaboration to address the challenges of these long and complex cancer histories.

While patients underwent MRRT, their treatment intent changed continuously from curative to palliative. Whereas the first RT course was given with a curative intent in about half of the patients, it was surprising that a relevant proportion of our patients treated with MRRT remained at a curative intent for a longer period: 18.8% and 11.6% of the 3rd and 5th courses of RT, respectively, were coded as being curative. The treatment intent had been recorded at the time of the patient consultation by the responsible clinician using standard definitions of curative and palliative intent. However, situations where all (oligo-) metastases were treated with radical intent, were classified as “curative” according to the concepts of repeat and induced oligometastatic disease [13].

With substantially improved OS in cohorts of metastatic cancer patients, it is obvious that the differentiation between curative and palliative becomes blurry: e.g., RT for brain metastases has traditionally been considered as highly palliative, with OS times in the range of weeks or months [14, 15]. However, metastatic melanoma patients treated with double immune checkpoint inhibition and metastatic NSCLC patients treated with anaplastic lymphoma kinase (ALK)-targeting tyrosine kinase inhibitors (TKIs) are today achieving median OS beyond 3–5 years [16–18]. Such patients might still be considered “palliative”; however, the design of their personalized treatment strategies needs to take (very) long-term survival into account and therefore adopt the principles of treatment strategies traditionally considered as curative. We have previously introduced a so-called “dynamic oligometastatic state model”, where patients might change repetitively between oligometastatic and polymetastatic disease states depending on their patterns of response and failure to local and systemic therapy [13]. Patients as reported in our study and concepts as described above show the limitations of the traditional and binary concept of curative and palliative intent and the model of a linear patient journey from curative to palliative.

This observation of curative RT intent even at later stages of a malignant disease is supported by the favorable long-term OS in the patients treated with MRRT. Median OS from the date of primary cancer diagnosis for the total cohort was 6.1 years and 5-years OS was 57.3%. Median OS from the 1st RT course for the total cohort was 3.3 years and 5-years OS was 32.7%. With a median interval of 26.6 months between the 1st and 5th course of RT, OS measured from the 5th RT course was still favorable with a median of 1.3 years and 5-years OS of 15.7%. Consequently, these data show that the decision-making process continuously and successfully identified patients with a sufficient life expectancy to achieve a potential benefit of MRRT. Though prone to selection bias, patients treated with more than five compared to five courses of repeat RT were characterized by improved OS measured form the first RT course, with no difference in OS measured from the date of primary diagnosis. This indicates that continuous and consistent use and integration of RT into cancer care of long-term cancer patients might even contribute to their favorable survival.

To our knowledge, this analysis is the first which studied a patient series having undergone MRRT. However, the retrospective nature of our study and associated, well-documented limitations need to be considered. This is especially true with respect to patient selection as the study focused on 112 patients treated over a period of 10 years. Appropriate patient selection criteria need to be identified and prospectively validated. Nevertheless, 67.0% (n = 75) of the 112 patients with minimum five courses of RT were treated within the last 3 years of the study period, indicating the growing clinical relevance.

*In conclusion,* an increasing number of patients is treated with MRRT throughout their long-term cancer survivorship, indicating that RT will become a more important component within the concept of cancer as a chronic disease. Further research efforts are required to better understand the safety and efficacy profile of MRRT.

## Data Availability

Data from electronic patient records cannot be disclosed.

## Appendix

See Figs. 4, 5.

## Abbreviations

ALK: Anaplastic lymphoma kinase
BASEC: Business Administration System for Ethics Committees
FDG-PET: Fluorodeoxyglucose positron emission tomography
Gy: Gray
ICD-10: International classification of diseases, 10th edition
MRRT: Multiple repeat radiotherapy
NSCLC: Non-small cell lung cancer
OS: Overall survival
PFS: Progression-free survival
RT: Radiotherapy
SBRT: Stereotactic body radiotherapy
SRS: Stereotactic radiosurgery
TKIs: Tyrosine kinase inhibitors
USZ: University Hospital Zurich
